# Two Cases of BCG Osteomyelitis Diagnosed Through Polymerase Chain Reaction/Electrospray Ionization-Mass Spectrometry Technology

**DOI:** 10.1093/cid/ciy105

**Published:** 2018-02-22

**Authors:** Christina Spyridou, Hany Ragab, Ronan A Murphy, Anna Riddell, Mark Wilks, Michael R Millar, Andrew J Prendergast

**Affiliations:** 1 Royal London Children’s Hospital, Barts Health NHS Trust; 2 National Heart and Lung Institute, Imperial College London; 3 Department of Infection, Barts Health NHS Trust, London; 4 Blizard Institute, Queen Mary University of London, United Kingdom

To the Editor—We echo the views of Ozenci et al. [1] that polymerase chain reaction/electrospray ionization-mass spectrometry (PCR/ESI-MS) has value for broad microbial detection. We used PCR/ESI-MS at our hospital until its withdrawal and here present 2 cases to illustrate its utility.

A 14-month-old boy presented with reduced use of the left arm, with no recent trauma or fever. His arm was held adducted, but the remainder of the examination was unremarkable. Magnetic resonance imaging (MRI) showed extensive marrow signal abnormality in the left humerus. He received intravenous co-amoxiclav and was discharged on oral co-amoxiclav. Twenty days into treatment, with no improvement, he was admitted for bone biopsy and intravenous ceftriaxone. Histopathology showed mild chronic inflammation with giant cells. Bone culture showed no growth. Analysis by PCR/ESI-MS unexpectedly revealed *Mycobacterium tuberculosis* (MTb) complex. There was no history of tuberculosis contact. A tuberculin skin test (TST) showed 20 mm induration, but an interferon-gamma release assay (IGRA) was negative. He initiated rifampicin, isoniazid, pyrazinamide, and ethambutol. Several weeks later, culture revealed *Mycobacterium bovis* BCG, and pyrazinamide was stopped. Investigations did not reveal an immunodeficiency, and he completed 1 year of treatment with good resolution of function and no growth arrest in the affected bone.

A 9-month-old boy presented with 4 weeks of pain, fever, and reduced movement at the left elbow. MRI revealed osteomyelitis of the proximal radius and septic arthritis of the left elbow. A joint washout was followed by intravenous co-amoxiclav. Culture of the joint fluid and synovium was sterile. Following good initial response to treatment, he was discharged on oral co-amoxiclav. Three weeks later he developed new swelling of the left elbow. Imaging showed subluxation of the proximal radio-ulnar joint and a collection within the olecranon fossa. Another washout was performed but still no organism was identified. Analysis by PCR/ESI-MS revealed MTb complex. There was no history of tuberculosis contact. A TST showed 16mm induration, but the IGRA was negative. He initiated rifampicin, isoniazid, pyrazinamide, and ethambutol. Several weeks later, culture revealed *Mycobacterium bovis* BCG, and pyrazinamide was stopped. Three months into treatment, he developed left wrist swelling; imaging showed extensive multi-focal osteomyelitis, and he initiated subcutaneous gamma-interferon (50 mcg/m^2^ thrice-weekly) with good response. A specific molecular defect in the IFN-gamma/IL-12/IL-23 axis could not be identified.

In summary, 2 children presented with unexpected BCG osteomyelitis, which is a rare complication of vaccination and presents nonspecifically, often leading to delays in diagnosis [2, 3]. PCR/ESI-MS enabled identification of MTb complex and initiation of anti-tuberculous therapy prior to traditional culture results. In young children, prompt initiation of definitive treatment may prevent long-term growth plate damage. Broad-range molecular testing is therefore a valuable technique, and it is regrettable that PCR/ESI-MS will no longer facilitate earlier microbial diagnosis. Although the authors may be correct in attributing the demise of Iridica at least partially to its cost ($200–300 per test) [1], the only equivalent test developed by Karius [4] costs about $2000, has a longer turnaround time and is currently neither Conformité Européene nor Food and Drug Administration approved.

## Notes

***Funding*.** This work was supported by Barts Charity [grant MGU0294] and the Wellcome Trust [grant 108065/Z/15/Z to A. J. P.].

***Potential conflicts of interest.*** All authors: No reported conflicts of interest. All authors have submitted the ICMJE Form for Disclosure of Potential Conflicts of Interest. Conflicts that the editors consider relevant to the content of the manuscript have been disclosed.
